# Risk factors and nomogram prediction model for pneumothorax after CT-guided coaxial biopsy combined with microwave ablation in ground-glass nodules

**DOI:** 10.1016/j.clinsp.2025.100750

**Published:** 2025-08-16

**Authors:** Canxing Wu, Yangtao Zhao, Mengjun Chen, Jun Ma

**Affiliations:** Longyan First Affiliated Hospital of Fujian Medical University, Department of Thoracic Surgery, Longyan, Fujian Province, China

**Keywords:** Ground-glass nodule, CT-guided percutaneous biopsy, Microwave ablation, Pneumothorax, Logistic regression

## Abstract

**Objective:**

To identify risk factors and develop a validated nomogram for predicting pneumothorax after CT-guided coaxial biopsy with synchronous Microwave Ablation (MWA) in Ground-Glass Nodules (GGNs).

**Methods:**

383 GGN patients were divided into a training set (*n* = 268) and a validation set (*n* = 115) in a 7:3 ratio. Univariate and multivariate logistic regression were employed to identify risk factors, followed by the construction of a nomogram model. Receiver Operating Characteristic (ROC) curves and calibration plots were generated to evaluate model performance, with further validation in the independent cohort. Decision curve analysis was applied to assess clinical utility.

**Results:**

Pneumothorax occurred in 72 cases (26.87 %) in the training set and 32 cases (27.83 %) in the validation set. Multivariate logistic regression revealed that BMI, lesion location, lesion depth, needle diameter, and number of punctures were independent risk factors for pneumothorax (all *p* < 0.05). Factor importance ranking was as follows: number of punctures > BMI > lesion depth > lesion location > needle diameter. The nomogram demonstrated robust calibration and predictive accuracy, with C-index values of 0.877 (training set) and 0.897 (validation set). The areas under the ROC curve (AUC) were 0.875 (95 % CI: 0.825–0.926) and 0.897 (95 % CI: 0.829–0.965), respectively. Sensitivity and specificity were 0.855/0.813 (training set) and 0.765/0.823 (validation set).

**Conclusion:**

Key determinants of postprocedural pneumothorax in GGN patients were identified through logistic regression and nomogram modeling. The validated predictive model exhibited excellent discriminative ability and clinical applicability, providing a scientific basis for individualized risk assessment and intervention strategies.

## Highlights

• The present work identifies modifiable risk factors (e.g., needle diameter, puncture frequency) and patient-specific predictors (BMI, lesion depth) to optimize procedural safety.
• Methodological Rigor: The authors developed and validated a nomogram model (AUC: 0.875–0.897) using multicenter retrospective data (*n* = 383), incorporating Decision Curve Analysis (DCA) to confirm clinical utility.
• Practical Impact: The model enables preoperative risk stratification, aiding surgeons in tailoring interventions to reduce pneumothorax rates (26.8 % in the studied cohort) and improve patient outcomes.

## Introduction

Ground-Glass Nodules (GGNs), as common pulmonary lesions, have demonstrated a continuously increasing detection rate in clinical practice with the widespread application of thoracic Computed Tomography (CT) examinations.^1^ GGNs exhibit diverse pathological characteristics, ranging from benign inflammatory changes and adenomatous hyperplasia to early-stage lung adenocarcinoma manifestations.^2^ Accurate discrimination between benign and malignant GGNs, followed by effective therapeutic interventions, is of paramount significance for improving patient prognosis.^3^

The concurrent CT-guided coaxial cannula puncture biopsy with Microwave Ablation (MWA) technique has consequently emerged as an integrated diagnostic-therapeutic approach. This modality enables simultaneous acquisition of pathological specimens for definitive diagnosis and immediate in situ inactivation of malignant lesions, offering distinct advantages including minimal invasiveness, rapid recovery, and confirmed therapeutic efficacy, thereby providing an efficient management strategy for GGN patients.^4^

However, this technique faces clinical challenges, with pneumothorax representing the most prevalent complication. Pneumothorax occurrence not only prolongs hospitalization duration and increases medical expenditures but may also impair respiratory function or even become life-threatening in severe cases. Current clinical studies report pneumothorax incidence rates ranging between 15 %‒30 % following this combined procedure in GGN patients.^5^ Such considerable incidence rates have engendered clinician reservations regarding treatment selection and imposed potential risks on patients.

Presently, no definitive consensus exists within the academic community regarding risk factors influencing pneumothorax development after CT-guided coaxial cannula biopsy with concurrent MWA in GGN patients.^6^ Clinical practice notably lacks precise predictive models for identifying high-risk individuals, resulting in the inability to formulate personalized preventive strategies targeting risk factors preoperatively. Suboptimal intraprocedural technique modification for pneumothorax risk mitigation. Consequently, restricted widespread adoption and further therapeutic efficacy enhancement of this technique. Against this background, the present study systematically screened pneumothorax risk factors through logistic regression analysis, performed factor prioritization, and subsequently developed and validated a nomogram prediction model. This study proposes to establish a validated predictive tool to enhance the clinical implementation of CT-guided coaxial biopsy with synchronous MWA for GGNs. The developed model enables preoperative risk stratification to identify high-risk patients, facilitates procedure optimization through evidence-based protocol modifications, and aims to significantly reduce pneumothorax complications. By improving both the safety profile and therapeutic efficacy of this combined diagnostic-interventional approach, these findings may contribute to standardized practice guidelines and promote wider adoption of this technique in the precision management of pulmonary GGNs.

## Materials and methods

### Study subjects

Patients with GGNs who underwent CT-guided coaxial cannula puncture biopsy and simultaneous MWA in the hospital from January 2022 to December 2024 were selected as the study subjects. Inclusion criteria were as follows: a single GGN was shown by chest CT examination, with a diameter ≤ 3 cm; the nodules were clearly diagnosed as benign or malignant by pathological diagnosis; the age was ≥ 18 years; the clinical data were complete. Exclusion criteria were as follows: complicated with severe dysfunction of important organs such as the heart, liver, and kidney; abnormal coagulation function; a history of previous thoracic surgery or severe pulmonary diseases (such as extensive distribution of pulmonary bullae and severe pulmonary fibrosis); inability to cooperate in completing puncture and ablation treatment. The patients were randomly divided into a training set (*n* = 268) and a validation set (*n* = 115) at a ratio of 7:3 using the complete randomization method. All methods were carried out in accordance with relevant guidelines and regulations. All experimental protocols were approved by the Ethics Committee of Longyan First Hospital.

### Data collection

Clinical data of patients were collected through the electronic medical record system, including: (1) General information: age, gender, BMI, smoking history (≥ 10 packs/year), underlying diseases (emphysema, pulmonary fibrosis, hypertension, diabetes); (2) Lesion characteristics: lesion location (upper lobe, middle lobe, lower lobe), lesion depth (distance from the lesion to the pleural surface, mm); (3) Operation-related parameters: diameter of the puncture needle (18 G, 20 G, 22 G), number of punctures (single, multiple), whether passing through the interlobar fissure or pulmonary bulla, ablation power (W), ablation time (min), indwelling time of the coaxial cannula (min).

### Grouping criteria for the training set

Whether pneumothorax occurred after surgery (definitely diagnosed by chest X-Ray or CT) was used as the dependent variable. Patients with pneumothorax were included in the pneumothorax group, while those without pneumothorax were included in the non-pneumothorax group.

### Statistical analysis

Statistical analysis was performed using SPSS 26.0 and R language 4.5.3. Count data were presented as the number of cases (percentage), and the χ^2^ test was used for comparison between groups. Measurement data conforming to the normal distribution were expressed as ±*s*, and the independent-samples *t*-test was used for comparison between groups. Multivariate Logistic regression analysis was used to screen risk factors, and a difference was considered statistically significant when *p* < 0.05. In the *R* language, the “rms” package was used to construct a nomogram model. The Bootstrap method was adopted to conduct internal validation of the model, and the calibration curve of the predicted results and the actual results was plotted. The Concordance-index (C-index) of the model was calculated, and the Hosmer-Lemeshow test was used to evaluate the goodness-of-fit of the prediction model. Decision Curve Analysis (DCA) was used to evaluate the clinical application value of the model.

## Results

### Comparison of general clinical characteristics of patients in the training set and the validation set

No statistically significant differences were found between the two groups in terms of indicators such as age, gender, BMI, smoking history, underlying diseases, lesion location, lesion depth, puncture needle diameter, and number of punctures (*p* > 0.05), Table 1.

**Table 1:** Comparison of general clinical characteristics of patients in the training set and the validation set.

Indicators		Training set (*n* = 268)	Validation set (*n* = 115)	χ²/*t*	p
Age (years)		57. 61 ± 8.81	56. 88 ± 9.01	0.591	0.555
Sex (Male/Female)		147/121 (54.85/45.15)	60/55 (52.17/47.83)	0.232	0.629
BMI (kg/m^2^)		21. 56 ± 3.02	21. 88 ± 2.99	0.775	0.439
Smoking history (≥10 packs/year)		100/168 (37.31/62.69)	50/65 (43.48/56.52)	1.284	0.257
History of emphysema		70/198 (26.12/73.88)	40/75 (34.78/65.22)	2.950	0.085
History of pulmonary fibrosis		40/228 (14.92/85.07)	20/95 (17.39/82.61)	0.370	0.543
History of hypertension		119/149 (44.40/55.60)	52/63 (45.21/54.79)	0.022	0.883
History of diabetes		50/218 (18.65/81.35)	30/85 (26.08/73.91)	2.688	0.101
Lesion location (Upper lobe/Middle lobe/Lower lobe)	Upper lobe	148 (55.22)	64 (55.65)	0.006	0.997
	Middle lobe	52 (19.40)	22 (19.13)		
	Lower lobe	68 (25.38)	29 (25.22)		
Lesion depth (mm)		33.28±11.51	35.61±10.55	1.563	0.119
Puncture needle diameter (G)	18G	78 (29.10)	35 (30.43)	0.215	0.898
	20G	114 (42.54)	50 (43.48)		
	22G	76 (28.36)	30 (26.09)		
Number of punctures	Single	136 (50.74)	58 (50.43)	0.003	0.956
	Multiple	132 (49.26)	57 (49.57)		
Passage through interlobar fissure (Yes/No)		48/220 (17.91/82.09)	20/95 (17.39/82.61)	0.015	0.903
Passage through pulmonary bulla (Yes/No)		53/215 (19.77/80.23)	30/85 (26.09/73.91)	1.888	0.169
Ablation power (W)		50. 22 ± 6.62	49. 66 ± 6.88	0.596	0.551
Ablation time (min)		11. 52 ± 3.11	11. 21 ± 2.89	0.763	0.447
Indwelling time of coaxial cannula (min)		14. 01 ± 4.22	13. 01 ± 3.68	1.894	0.059

### Univariate analysis of pneumothorax after synchronous MWA treatment combined with CT-guided coaxial cannula puncture biopsy in patients with GGNs in the training set

In the training set, there were 72 cases in the pneumothorax group and 196 cases in the non-pneumothorax group. Univariate analysis showed that there were statistically significant differences in Body Mass Index (BMI), smoking history, history of emphysema, history of pulmonary fibrosis, lesion location, lesion depth, diameter of the puncture needle, number of punctures, and passage through bullae between the two groups (*p* < 0.05), Table 2.

**Table 2:** Univariate analysis of pneumothorax after synchronous MWA treatment combined with CT-guided coaxial cannula puncture biopsy in patients with GGNs in the training set.

Indicators		Pneumothorax group (*n* = 72)	Non-pneumothorax group (*n* = 196)	χ^2^/t	p-value
Age (years)		57. 81 ± 8.51	55. 52 ± 8.96	1.879	0.061
Sex (Male/Female)		45/27 (62.50/37.50)	102/94 (52.05/47.95)	2.326	0.127
BMI (kg/m^2^)		20. 51 ± 3.22	22. 12 ± 2.89	3.918	0.001
Smoking history (≥10 packs/year)		40/32 (55.55/44.45)	60/136 (60.61/69.39)	14.006	0.001
History of emphysema		28/44 (38.88/61.12)	42/154 (21.43/78.57)	8.319	0.004
History of pulmonary fibrosis		20/52 (27.77/62.23)	20/176 (10.20/89.80)	12.807	0.001
History of hypertension		35/37 (48.61/51.39)	84/112 (42.86/57.14)	0.706	0.401
History of diabetes		18/54 (25.00/75.00)	32/164 (16.32/83.68)	2.611	0.106
Lesion location (Upper lobe/Middle lobe/Lower lobe)	Upper lobe	50 (69.44)	98 (50.00)	9.103	0.011
	Middle lobe	12 (16.66)	40 (20.41)		
	Lower lobe	10 (13.90)	58 (29.59)		
Lesion depth (mm)			30.61±10.35	3.071	0.002
Puncture needle diameter (G)	18G	30 (41.66)	48 (24.49)	8.405	0.015
	20G	28 (38.88)	86 (43.88)		
	22G	14 (19.46)	62 (31.63)		
Number of punctures	Single	25 (34.72)	111 (56.63)	10.114	0.002
	Multiple	47 (65.28)	85 (43.37)		
Passage through interlobar fissure (Yes/No)		18/54 (25.00/75.00)	30/166 (15.30/84.70)	3.366	0.066
Passage through pulmonary bulla (Yes/No)		22/50 (30.55/69.45)	31/165 (32.29/67.708)	7.211	0.007
Ablation power (W)		51. 31 ± 6.52	49. 76 ± 6.83	1.666	0.096
Ablation time (min)		11. 51 ± 3.21	10. 21 ± 2.88	3.175	0.002
Indwelling time of coaxial cannula (min)		14. 21 ± 4.82	13. 31 ± 3.62	1.643	0.102

### Multivariate logistic regression analysis

Multivariate Logistic regression analysis was performed with the occurrence of pneumothorax (1 = yes, 0 = no) as the dependent variable and the factors with *p* < 0.05 in the univariate analysis as covariates. The results showed that BMI, lesion location, lesion depth, puncture needle diameter, and number of punctures were independent influencing factors for the occurrence of pneumothorax (*p* < 0.05), Table 3.

**Table 3:** Multivariate Logistic regression analysis.

Factor	*β*	SE	Wald	p	OR	95 %CI
BMI	−0.292	0.067	18.922	0.001	0.747	0.655‒0.852
Lesion Location	−1.013	0.460	4.838	0.028	0.363	0.147‒0.896
Lesion Depth	0.083	0.020	18.135	0.001	1.087	1.046‒1.130
Puncture Needle Diameter	−1.646	0.506	10.590	0.001	0.193	0.072‒0.520
Number of punctures	−4.180	0.622	45.119	0.001	0.015	0.005‒0.052

### Ranking of the importance of influencing factors

**Fig. 1:**
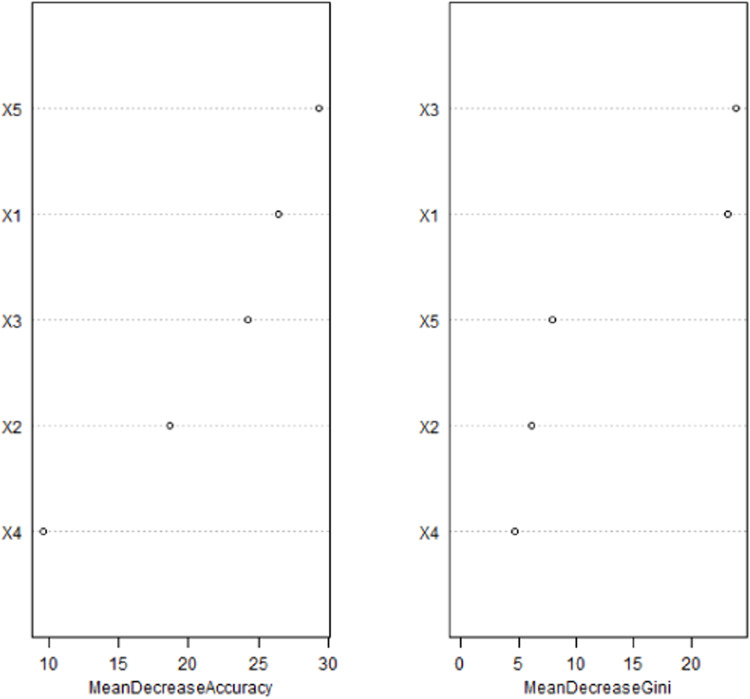
Ranking of the importance of influencing factors.

Based on the absolute values of the standardized regression coefficients of each influencing factor obtained from the multivariate Logistic regression analysis, the importance of the influencing factors was ranked (Table 4). The result was that the number of punctures > BMI > lesion depth > lesion location > puncture needle diameter, Fig. 1.

**Table 4:** Variable assignment methods.

Variables	Meanings	Assignments
X1	BMI	Continuous variable
X2	Lesion Location	0 = Upper lobe, 1 = Middle lobe, 2 = Lower lobe
X3	Lesion Depth	Continuous variable
X4	Puncture Needle Diameter	0 = 18 G, 1 = 20 G, 2 = 22G
X5	Number of punctures	0 = Multiple, 1 = Single
Y	Pneumothorax	0 = No, 1 = Yes

### Establishment of the nomogram prediction model

**Fig. 2:**
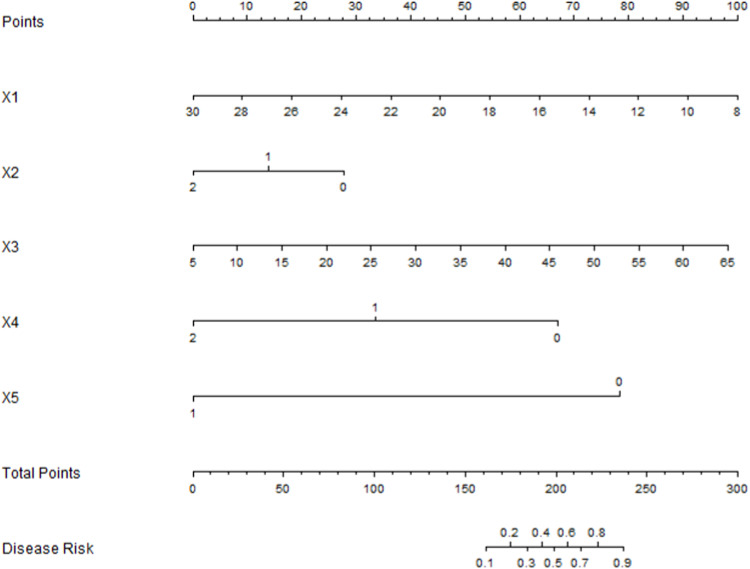
Nomogram model for pneumothorax after synchronous MWA treatment combined with CT-guided coaxial cannula puncture biopsy in patients with GGN. Note: X1, BMI; X2, Lesion location; X3, Lesion depth; X4, Puncture needle diameter; X5, Number of punctures.

Based on the results of the multivariate Logistic regression analysis, a Nomogram model was constructed to predict pneumothorax in patients with GGN after simultaneous MWA treatment via CT-guided coaxial cannula puncture biopsy. Scores were assigned to each independent risk factor in the model, and the total score was obtained by adding up the scores of each factor. Through the total score, the predicted probability of caregivers' demand for “respite care” could be obtained from the Nomogram model (Fig. 2).

### Evaluation and validation of the nomogram model for pneumothorax after synchronous MWA treatment combined with CT-guided coaxial cannula puncture biopsy in patients with GGN

**Fig. 3:**
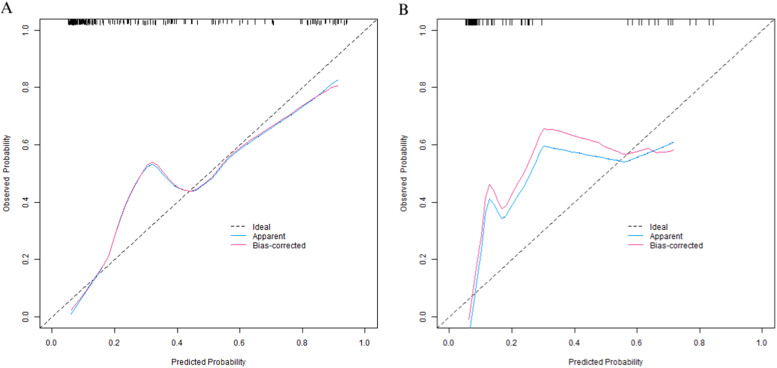
Calibration curves are presented (Curve A represents the calibration curve for the training set, while Curve B represents that for the validation set).

**Fig. 4:**
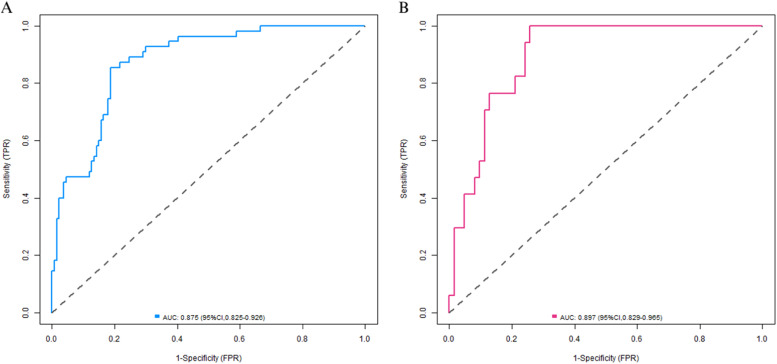
ROC curves (A is the ROC curve of the training set, B is the ROC curve of the validation set).

In the training set, the C-index of the nomogram prediction model was 0.877. The calibration curve showed that the mean absolute error of the agreement between the predicted values and the actual values was 0.128. The Hosmer-Lemeshow test yielded a p-value of 0.026, indicating a good model fit. The ROC curve showed that the AUC of the model for predicting early postoperative recurrence was 0.875 (95 % CI: 0.825‒0.926), with a sensitivity of 0.855 and a specificity of 0.813. In the validation set, the C-index was 0.897, the mean absolute error was 0.120, the Hosmer-Lemeshow test p-value was 0.314, the AUC was 0.897 (95 % CI: 0.829‒0.965), the sensitivity was 0.765, and the specificity was 0.823. The calibration curve and the ROC curve are shown in Fig. 3, Fig. 4, respectively.

### Decision curve analysis of the nomogram prediction model

**Fig. 5:**
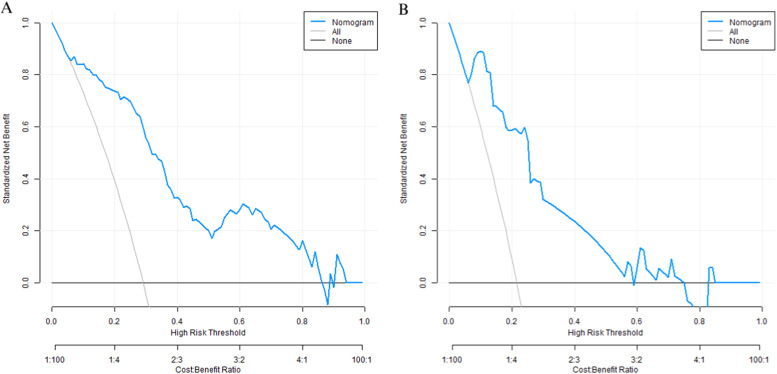
Decision curves (A represents the decision curve of the training set, B represents the decision curve of the validation set).

The decision curve shows that when the threshold probability is between 0.10 and 0.80, the decision predicted by the nomogram model constructed in this study has more clinical benefits (Fig. 5).

## Discussion

With the popularization of chest CT examinations, the detection rate of Ground-Glass Nodules (GGNs) has increased significantly. As a common subsolid pulmonary lesion, GGNs have diverse natures. They may be benign hyperplasia or inflammation, or manifestations of early-stage lung cancer. Therefore, accurately distinguishing their benign and malignant natures and implementing timely treatment are crucial for improving patients' prognoses.^7^ The technique of CT-guided coaxial cannula puncture biopsy combined with simultaneous MWA has become an important means for the diagnosis and treatment of GGNs because it can simultaneously complete pathological diagnosis and in-situ treatment, with the advantages of minimal invasiveness and high efficiency.^8^ However, pneumothorax, one of the most common complications of this technique, has an incidence rate as high as 15 %‒30 %. It not only increases patients' pain and medical burden but may also endanger life in severe cases, which restricts the wide application of this technique.^9^ Currently, there is no clear consensus in the academic community regarding the influencing factors of pneumothorax in GGN patients after this combined treatment. Clinically, there is a lack of an accurate prediction model to identify high-risk patients. Existing studies mostly focus on single-factor analysis, and the conclusions of different studies vary. For example, the roles of factors such as the diameter of the puncture needle and the location of the lesion have not been fully clarified. Therefore, based on large-sample retrospective data, this study systematically screened the independent influencing factors of pneumothorax through Logistic regression and the Nomogram model and ranked their importance. The aim was to construct an efficient and operable prediction model to provide a scientific basis for preoperative risk assessment, optimization of operation plans, and individualized intervention in clinical practice, thereby reducing the incidence of pneumothorax and improving the safety of treatment.

In this study, through univariate and multivariate Logistic regression analyses, five independent risk factors were screened out from 16 potential influencing factors: BMI, lesion location, lesion depth, puncture needle diameter, and number of punctures. And their importance was ranked according to the absolute value of the standardized regression coefficient as follows: number of punctures > BMI > lesion depth > lesion location > puncture needle diameter. This result not only clarifies the differences in the contributions of each factor to the occurrence of pneumothorax but also provides a basis for clinical priority intervention. In terms of model construction, the Nomogram integrates the five independent factors in a visual way, converting the risk contributions of each variable into specific scores, which facilitates clinicians to quickly calculate the probability of pneumothorax in patients. The validation results of the training set and the validation set showed that the model had good predictive performance and calibration: the C-index of the training set was 0.877, and that of the validation set was 0.897, both higher than 0.85, indicating excellent discrimination of the model; the area under the ROC Curve (AUC) was 0.875 and 0.897 respectively, and both the sensitivity and specificity exceeded 75 %, suggesting high accuracy of the model in identifying high-risk patients. The calibration curve showed a high degree of agreement between the predicted probability and the actual incidence. The p-values of the Hosmer-Lemeshow test were all greater than 0.05 (*p* = 0.314 in the validation set), further verifying the goodness-of-fit of the model. Decision curve analysis (DCA) indicated that when the threshold probability was between 0.10‒0.80, the clinical benefit of the model was significantly better than the “treat – all” or “treat – none” strategies, suggesting high application value of the model in actual clinical scenarios. It is worth noting that in this study, the training set and the validation set were divided at a ratio of 7:3, and internal validation was carried out by the Bootstrap method, which reduced the risk of overfitting. However, external validation of the model has not been carried out, which is related to the single-center retrospective design of the study. Further multicenter prospective studies are needed to verify the universality of the model.

The number of punctures ranked first among the influencing factors (OR = 0.015, *p* < 0.001), suggesting that multiple punctures significantly increase the risk of pneumothorax.^10^ This may be related to the repeated damage to the visceral pleura during the puncture process: each puncture may cause a pleural tear, and multiple punctures make it difficult for the tear to repair, allowing air to enter the thoracic cavity more easily.^11^ In addition, repeated adjustment of the puncture path may exacerbate lung tissue damage, especially when passing through the interlobar fissure or pulmonary bullae, further increasing the risk of pneumothorax. In clinical practice, the number of punctures should be reduced by accurately planning the puncture path before the operation and improving the success rate of the first puncture. For example, three-dimensional reconstruction technology can be used to optimize the needle-entry angle to avoid blind puncture.^12^ BMI was negatively correlated with the occurrence of pneumothorax (OR = 0.747, *p* = 0.001); that is, the lower the BMI, the higher the risk of pneumothorax. Patients with low BMI may have poor lung tissue elasticity and a thinner subpleural fat layer. After a puncture, the pleural tear is not easy to close, and the low surface tension of the lung leads to continuous gas leakage.^13^ In addition, thin patients may have underlying interstitial lung diseases (such as pulmonary fibrosis). Although patients with severe pulmonary fibrosis were excluded in the exclusion criteria of this study, mild interstitial changes may not have been completely excluded, which may indirectly affect the results.^14^ Clinically, for patients with low BMI, the risk of pneumothorax should be vigilant. Prolonged postoperative observation time or preventive closed - chest drainage can be considered. Lesion depth was positively correlated with the risk of pneumothorax (OR = 1.087, *p* < 0.001), that is, the farther the lesion is from the pleural surface, the higher the incidence of pneumothorax.^15^ The puncture path for deep-seated lesions is longer and needs to pass through more lung tissue, increasing the probability of damage to the lung parenchyma and the pleura.^16^ In addition, the positioning of deep-seated lesions is more difficult, which may lead to an increase in the number of punctures or repeated adjustments of the direction, further exacerbating the damage.^17^ For deep-seated GGNs, it is recommended to use a thinner puncture needle (such as 22 G) to reduce tissue damage, and pay attention to controlling the power and time during the ablation process to avoid excessive thermal damage that increases the brittleness of the lung tissue. The risk of pneumothorax in patients with lesions located in the upper lobe was significantly higher than that in the middle and lower lobes (OR = 0.363, *p* = 0.028), which may be related to the anatomical location of the upper lobe: the upper lobe has a larger volume, and its surface is closer to the chest wall. During puncture, the exposed area of the pleura is large, and the upper-lobe lung tissue has a higher range of motion during breathing, which is easy to rub against the chest wall and cause the tear to expand.^18^ In addition, for upper-lobe lesions, the needle often needs to be inserted from the lateral chest wall, and the puncture path may pass through more pulmonary bullae (univariate analysis in this study showed that passing through pulmonary bullae was related to pneumothorax). The thin-walled pulmonary bullae are easy to rupture, increasing the risk of air leakage.^19^ Clinically, for upper-lobe lesions, the supine or lateral position can be preferentially selected, and body position adjustment can be used to reduce lung tissue movement and avoid areas with dense pulmonary bullae. The puncture needle diameter was negatively correlated with the risk of pneumothorax (OR = 0.193, *p* = 0.001), that is, the thicker the needle diameter, the higher the risk.^20^ The risk of pneumothorax with an 18 G needle was significantly higher than that with 20 G and 22 G needles because the physical damage to the lung tissue and the pleura is greater when using a thick needle. The diameter of the tear is positively correlated with the needle diameter, resulting in an increase in the amount of gas leakage. .^21^ Although a thick needle may improve the success rate of biopsy, a balance needs to be struck between the diagnostic needs and the risk of complications.^22^ For patients at high risk of pneumothorax (such as those with low BMI and upper-lobe lesions), it is recommended to preferentially choose a 22-G thin needle to reduce damage while ensuring pathological sampling.^23^

This study has certain limitations. First, as a single-center retrospective study, the sample source is single, and there may be selection bias. For example, all the included patients are from the same medical center, and the operation specifications and equipment models are relatively uniform. Differences between different institutions need to be considered when promoting the model externally. Second, external validation was not carried out. Although internal validation showed good model performance, the prediction ability under different populations and different equipment conditions is not clear, which is mainly limited by the lack of data sharing in current similar studies and the time cost of multicenter collaboration. In addition, some potential factors such as the intensity of the patient's cough reflex and the breathing control during puncture were not included in the analysis, which may have a certain impact on the results. Future studies can be expanded in the following directions: Conduct multicenter prospective studies, including patients under different geographical and equipment conditions, to verify the external validity of the model; Combine imaging features (such as the internal density and marginal morphology of GGNs) and molecular biological indicators to further optimize the predictive performance of the model; Develop intervention measures for high-risk factors (such as operation specifications for reducing the number of punctures and pretreatment plans for patients with low BMI) and conduct randomized controlled trials to evaluate the preventive effect.

In conclusion, this study identified five independent influencing factors of pneumothorax in GGN patients after CT-guided puncture biopsy combined with simultaneous MWA treatment through large-sample analysis and constructed an efficient Nomogram prediction model. In clinical practice, high-risk populations can be quickly identified by evaluating the patient's BMI, lesion location and depth, puncture needle diameter, and number of punctures, and the operation strategy can be optimized accordingly (such as choosing a thin needle, reducing the number of punctures, and avoiding areas with dense pulmonary bullae), thereby reducing the incidence of pneumothorax. In the future, further multicenter studies are needed to verify the model and explore individualized preventive measures to promote the safe application of this technique.

## Data availability

The datasets used and/or analyzed during the current study are available from the corresponding author on reasonable request.

## Funding

This work was supported by the Longyan City Science and Technology Plan (No2023LYF17060). Please confirm this statement will be included in the final published article.

## Declaration of competing interest

The authors declare no conflicts of interest.
